# Models for synthetic biology

**DOI:** 10.1186/1752-0509-1-47

**Published:** 2007-11-06

**Authors:** Yiannis N Kaznessis

**Affiliations:** 1 Department of Chemical Engineering and Materials Science, University of Minnesota, Minneapolis, MN 55455, USA

## Abstract

Synthetic biological engineering is emerging from biology as a distinct discipline based on quantification. The technologies propelling synthetic biology are not new, nor is the concept of designing novel biological molecules. What is new is the emphasis on system behavior.

The objective is the design and construction of new biological devices and systems to deliver useful applications. Numerous synthetic gene circuits have been created in the past decade, including bistable switches, oscillators, and logic gates, and possible applications abound, including biofuels, detectors for biochemical and chemical weapons, disease diagnosis, and gene therapies.

More than fifty years after the discovery of the molecular structure of DNA, molecular biology is mature enough for real quantification that is useful for biological engineering applications, similar to the revolution in modeling in chemistry in the 1950s. With the excitement that synthetic biology is generating, the engineering and biological science communities appear remarkably willing to cross disciplinary boundaries toward a common goal.

Synthetic biological engineering is emerging from biology as a distinct discipline based on quantification [1-5]. The objective is the design and construction of new biological devices and systems to deliver useful applications. Numerous synthetic gene circuits have been created in the past decade, including bistable switches, oscillators, and logic gates [[1-5], and references therein], and possible applications abound, ranging from biofuels, to detectors for biochemical and chemical weapons, to disease diagnosis, to gene therapies.

Certainly, the technologies propelling synthetic biology are not new, nor is the concept of designing novel biological molecules [6,7]. What is perhaps new is the emphasis on system behavior, designing DNA sequences with synthetic phenotypes exhibiting prescribed dynamic responses.

Despite the initial successes of synthetic designs [1-5], the paradigm of biological sciences as descriptive disciplines may not rapidly assist in rationally engineering novel gene networks, despite the increasing volume of components that can be used in constructing synthetic networks. Genome projects identify the components of gene networks in biological organisms, gene after gene, and DNA microarray experiments discover the network connections. Yet, the static pictures of networks these experiments provide cannot adequately explain biomolecular phenomena or enable rational engineering of dynamic gene expression regulation. In other words, as an engineering discipline, synthetic biology cannot rely on endless trial and error methods driven by verbal description of biomolecular interaction networks.

The challenge facing the scientific and engineering communities is then to reduce the enormous volume and complexity of biological data into concise theoretical formulations with predictive ability, ultimately associating synthetic DNA sequences to dynamic phenotypes. The paradigm is not new either: In the 1940s and 1950s chemistry was a well matured discipline for pioneers like Neil Amundson, Byron Bird and Rutherford Aris to develop mathematical models that captured the enormous complexity of chemical processes in a way useful for chemical engineering applications [8-10]. Quantitative models of chemical processes led to the establishment of the chemical engineering discipline and the emergence of a strong chemical/petroleum industry. Although arguments can be made about the detrimental role of this industry on the environment, there can be no doubt of the overall positive effects on human life.

But what types of models are appropriate for synthetic biology? Because of the large number of participating species and the complexity of their interactions, only detailed modeling can allow the investigation of dynamic gene expression in a way fit for analysis and design. Designs can be detailed at the molecular level with dynamic models of all the biomolecular interactions involved in transcription, translation, regulation, transport and induction. We contrast this to *a posteriori *modeling of synthetic networks. For example in their seminal 2000 paper [11], Gardner and co-workers developed a very elegant model that captures and explains the observed dynamic behavior of the bistable switch and provides additional insight in the biological mechanism. This formalism may abide well with Occam's razor, but cannot guide the choice of specific DNA sequences and their regulatory relations to achieve a bistable switch. More specifically, it will be challenging to use reduced models to choose, for example, between lactose, arabinose or tetracycline operators, or any one of dozens of their mutant variants, for building a new, different bistable switch.

In engineering, descriptive models that are succinct and lucid are appreciated, but the ones used will be at the level of design degrees of freedom. For example, Bernoulli's equation can explain the aerodynamic lift of an airplane, but modern aircraft design is based on simulations that include all the components of flight in detail. Turning to synthetic biology, model-driven rational engineering of synthetic gene networks is possible at two levels:

First, the level of network topologies, where biomolecules control the concentration of other biomolecules, e.g. DNA binding proteins regulate the expression of specific genes by either activation or repression. By combining simple regulatory interactions, such as negative and positive feedback and feed forward loops, one may create more intricate networks that precisely control the production of protein molecules, such as bistable switches, oscillators, and filters. In the laboratory, these networks can be created using existing libraries of regulatory proteins and their corresponding operator sites. The now classical example is the aforementioned bistable switch Gardner and co-workers built [11]: they connected two regulatory proteins repressing one another and this resulted in a bistable switch they could control. Another is the repressilator of Elowitz and Leibler [12]: three regulatory proteins repressing one another in a sequential loop resulted in oscillating concentration profiles.

Secondly, the level of molecular components, which describes the kinetics and strengths of biomolecular interactions within the system. Indeed, the dynamical behavior of the system is a complex function of the kinetic interactions of the components. By altering the characteristics of the components, such as DNA-binding proteins and their corresponding DNA sites, one can modify the system's dynamical behavior without modifying the network topology. In the laboratory, the DNA sequences that yield the desired characteristics of each component can be engineered to achieve the desired protein-protein, protein-RNA, or protein-DNA binding constants and enzymatic activities. For example, Alon and co-workers [13] showed how simple mutations on the DNA sequence of the lactose operon can result in widely different phenotypic behaviors.

Ultimately, the large number of variants (interaction topologies and strengths) for these two types of design degrees of freedom requires sophisticated computational modeling, since the cost of experimentally changing these components and the kinetics of their interactions can quickly become prohibitive. Computer simulations enable exhaustive searches of different network connectivities and molecular thermodynamic/kinetic parameters, greatly advancing the development of design principles that seek to simplify the complicated behavior of the network into a brief, usable framework.

All gene expression molecular level events can be represented with reactions. For any two molecular species A and B (proteins, DNA, RNA, signaling molecules, etc.) interacting in solution to form a complex A*B (e.g. a repressor protein and the corresponding DNA operator site) we can write

[A]aq+[B]aq⇌k−1k1[A∗B]aq
 MathType@MTEF@5@5@+=feaafiart1ev1aaatCvAUfKttLearuWrP9MDH5MBPbIqV92AaeXatLxBI9gBaebbnrfifHhDYfgasaacPC6xNi=xI8qiVKYPFjYdHaVhbbf9v8qqaqFr0xc9vqFj0dXdbba91qpepeI8k8fiI+fsY=rqGqVepae9pg0db9vqaiVgFr0xfr=xfr=xc9adbaqaaeGacaGaaiaabeqaaeqabiWaaaGcbaGaei4waSLaeeyqaeKaeiyxa01aaSbaaSqaaiabbggaHjabbghaXbqabaGccqGHRaWkcqGGBbWwcqqGcbGqcqGGDbqxdaWgaaWcbaGaeeyyaeMaeeyCaehabeaakmaao4aaleaacqWGRbWAdaWgaaadbaGaeeymaedabeaaaSqaaiabdUgaRnaaBaaameaacqGHsislcqqGXaqmaeqaaaGccaGLahIaayzVHaGaei4waSLaeeyqaeKaey4fIOIaeeOqaiKaeiyxa01aaSbaaSqaaiabbggaHjabbghaXbqabaaaaa@4B4F@

with k_1 _and k_-1 _the association and dissociation kinetic constants, respectively. If we considered the cell as a well-stirred reactor we could calculate the behavior of the network using a set of ordinary differential equations, which determine concentration changes as prescribed by kinetic laws. However, the underlying assumption of such continuous-deterministic models, that the number of molecules approaches the thermodynamic limit (i.e. that the volume of the system is infinite), can be invalid for biological systems, since for some components (DNA for example) there are only a few copies available.

In the 1950s Oppenheim and McQuarrie, among others, explored stochasticity in kinetic models, developing the chemical Master equation formalism to capture discrete interaction events that occur with certain probability in time [14,15]. A numerical stochastic simulation algorithm (SSA) to calculate these probabilistic trajectories was described by Gillespie [16]. Gillespie's algorithm uses the system dynamics to simulate the occurrence of each individual reaction event. In general, given the current state of the system, the SSA seeks the time until the next reaction occurs. It then executes that reaction, updates the state of the system, and increments the simulation time to the new value. Although accurate in capturing the dynamic of biomolecular interaction systems, SSA becomes computationally intractable, if the time scales of involved interaction events are disparate, because it simulates every single biomolecular interaction event, spending inordinate amounts on fast reactions for very few simulated occurrences of slow reactions. The modeling community was up to the challenge and in the last decade there have been numerous attempts to improve the efficiency of the SSA [17-23]. As a result, recently algorithms have appeared that successfully tackle biomolecular interaction phenomena with disparate time scales [24-29] (see Figure 1). Although work is still underway, there are now exciting developments that the synthetic biology community can benefit from.

**Figure 1:**
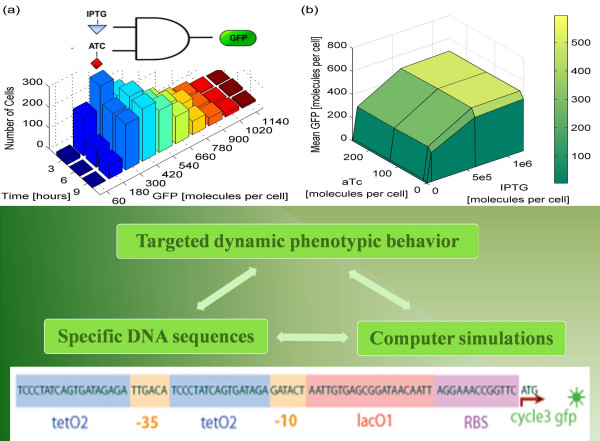
**A major challenge in synthetic biology is to rationally select DNA sequences that result in targeted dynamic phenotypes**. For example, with simulations using Hy3S [29] we are experimenting with multiple alternative promoter sequences to identify the optimal AND gate synthetic gene network, with tetracycline (atc) and IPTG as inputs and green fluorescence protein (GFP) as output.

More than fifty years after the discovery of the molecular structure of DNA, molecular biology is mature enough for quantification useful for biological engineering applications, similar to chemistry in the 1950s. With the excitement synthetic biology is generating, the engineering and biological science communities appear remarkably willing to cross disciplinary boundaries toward this common goal.
